# Supplementary material for: Emergence of Topological insulator and Nodal line semi-metal states in XX′Bi (X = Na, K, Rb, Cs; X′ = Ca, Sr)
Source: Sci Rep. 2019 Jan 24;9:527. doi: 10.1038/s41598-018-36869-0 (PMC6345778; doi:10.1038/s41598-018-36869-0)
Supplement: Supplementary file 1 — Supplementary Information [file 41598_2018_36869_MOESM1_ESM.pdf]

Supplementary Material

Emergence of Topological insulator and Nodal line semi-metal states in XX'Bi (X=Na, K, Rb, Cs; X'=Ca, Sr)

Chiranjit Mondal[1], C. K. Barman[2], Sourabh Kumar[1] ,Aftab Alam[2] and Biswarup Pathak[1]

[1]*Discipline of Metallurgy Engineering and Materials Science, IIT Indore, Simrol, Indore 453552, India*
[2]*Department of Physics, Indian Institute of Technology, Bombay, Powai, Mumbai 400 076, India*

In this supplementary material, we provide the detailed information of lattice parameter for all parent and doped systems . We have made a comparative analysis of bulk electronic structures using two different level of calculations (PBE vs HSE06). Detailed description of surface calculations are provided systematically. Both topological insulator and nodal line semi-metal states are discussed explicitly.

In order to investigate the possibilities of the experimental synthesis for the proposed compounds, we have checked the chemical stability of these compounds by calculating the formation energies.
In the Table-SI, we have listed calculated (using PBE functionals) lattice parameters and formation energies of all compounds. The formation energies are calculated using the formula , where E_{alloy} is the total energy of the compound and E_i represent the energy of the constituent elements in their equilibrium phases, all at their equilibrium lattice parameter. x_i is the proportion of the ith element in the compound. Negative formation energy supports the stability of these compounds.

I. Relaxed lattice parameters and formation energies

System	Relaxed lattice parameter (Å)		Formation energy (meV/atom)
	**a = b**	**c**	
NaCaBi	8.19	4.81	-680
$Na_{0.66}K_{0.33}CaBi$	8.31	4.88	-600
$Na_{0.33}K_{0.66}CaBi$	8.63	4.92	-560
$Na_{0.66}Rb_{0.33}CaBi$	8.37	4.92	-560
$Na_{0.33}Rb_{0.66}CaBi$	8.89	4.96	-500
$Na_{0.66}Cs_{0.33}CaBi$	8.43	4.96	-520
$Na_{0.33}Cs_{0.66}CaBi$	9.41	5.02	-470
NaSrBi	8.44	4.98	-740

$Na_{0.66}K_{0.33}SrBi$	8.54	5.05	-690
$Na_{0.33}K_{0.66}SrBi$	8.86	5.09	-650
$Na_{0.66}Rb_{0.33}SrBi$	8.60	5.08	-650
$Na_{0.33}Rb_{0.66}SrBi$	9.06	5.13	-600
$Na_{0.66}Cs_{0.33}SrBi$	8.66	5.12	-620
$Na_{0.33}Cs_{0.66}SrBi$	9.33	5.15	-560
KCaBi	8.88	5.00	-466
KSrBi	9.04	5.17	-587
RbCaBi	9.20	5.05	-340
RbSrBi	9.33	5.25	-480
CsCaBi	9.60	5.05	-230
CsSrBi	9.68	5.26	-380

Table SI. Calculated relaxed lattice parameters and formation energies for all parent and doped systems.

II. Dynamical Stability

To further check the dynamical stability, we calculated the phonon dispersion at T = 0 K for the three prototype compounds (NaCaBi, NaSrBi and RbCaBi). The phonon dispersions for the topologically non-trivial phase of NaCaBi and NaSrBi are calculated at ambient condition as well as at various bi-axial and hydrostatic pressure. For the RbCaBi, we have calculated phonon frequencies at equilibrium lattice parameter. The phonon dispersions are shown in Figure (S1-S3). 2×2×2 supercell having 72 atoms are used to calculate the force constants.

It is obvious from Fig. S1 & S2, that there exists no imaginary phonon frequency for NaCaBi and NaSrBi upto 5% strain (both BAS and HP), confirming the dynamical stability of these compounds. For the RbCaBi, the presence of small negative phonon frequencies at ambient condition (see Fig. S3) indicates the dynamical instability at T = 0K. The existence of such negative phonon frequencies give an indication of structural instabilities and hints a very flat energy profile among the various competing phase near the equilibrium structure. The negative phonon frequencies, however, often get stabilized at finite temperature calculations. Such scenario, for instance, is very common in perovskite family [1-6] There also exists other class of systems e.g MoS_2 where the first principles calculation at T=0 K [See Ref. *PRB 88, 245428 (2013)*] show imaginary phonon frequencies up to 280 cm^{-1}, but they are well know systems to be synthesized in the laboratory. As such we claim ~1.47 THz (50 cm^{-1}) imaginary frequency for RbCaBi is small enough and may not hinder the actual synthesis of this compound.

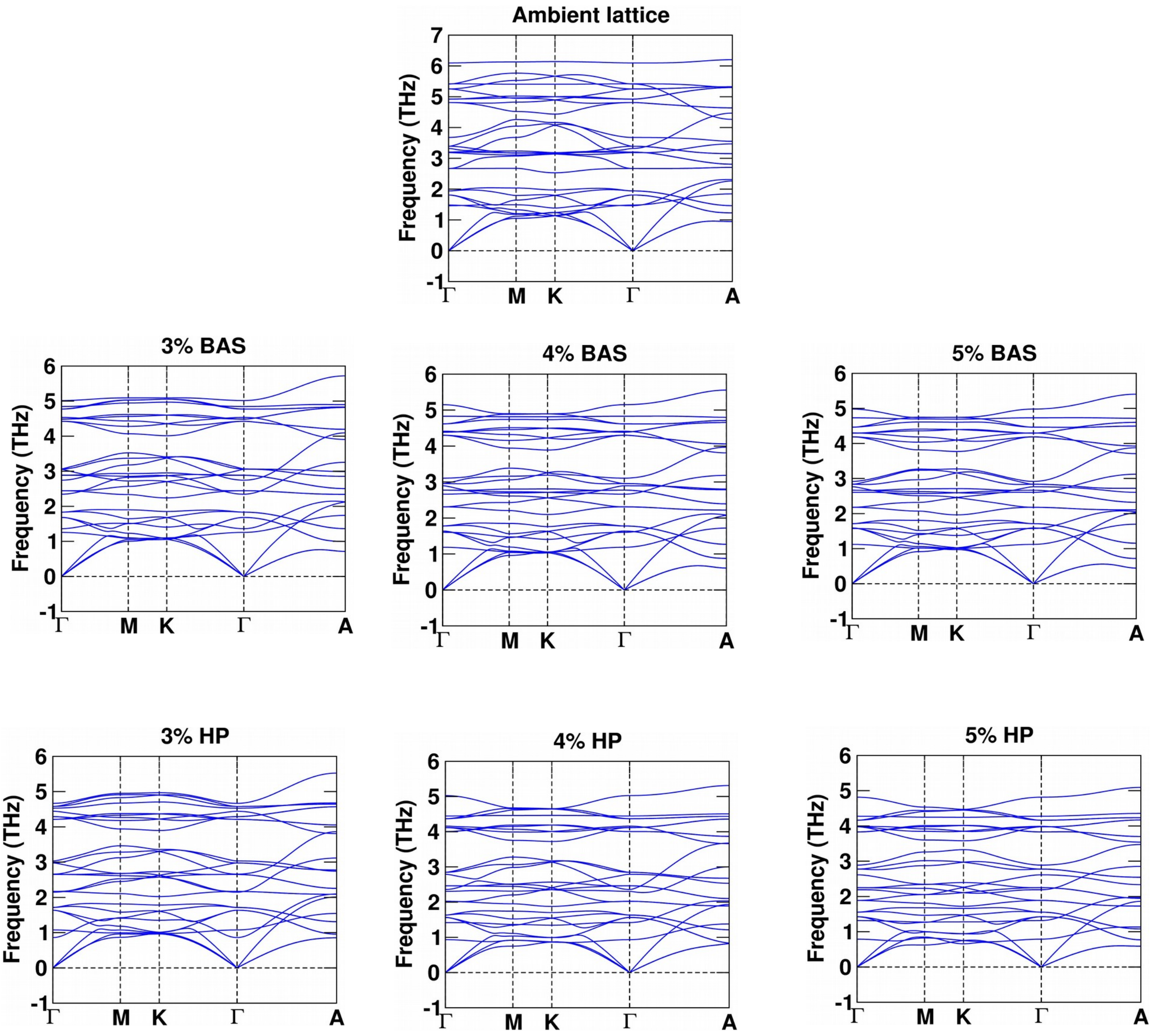

Fig. S1. Phonon dispersion of NaCaBi at (top-panel) ambient lattice constatnt, (midle-panel) various bi-axial strain (BAS) and (bottom-panel) hydrostatic pressure (HP).

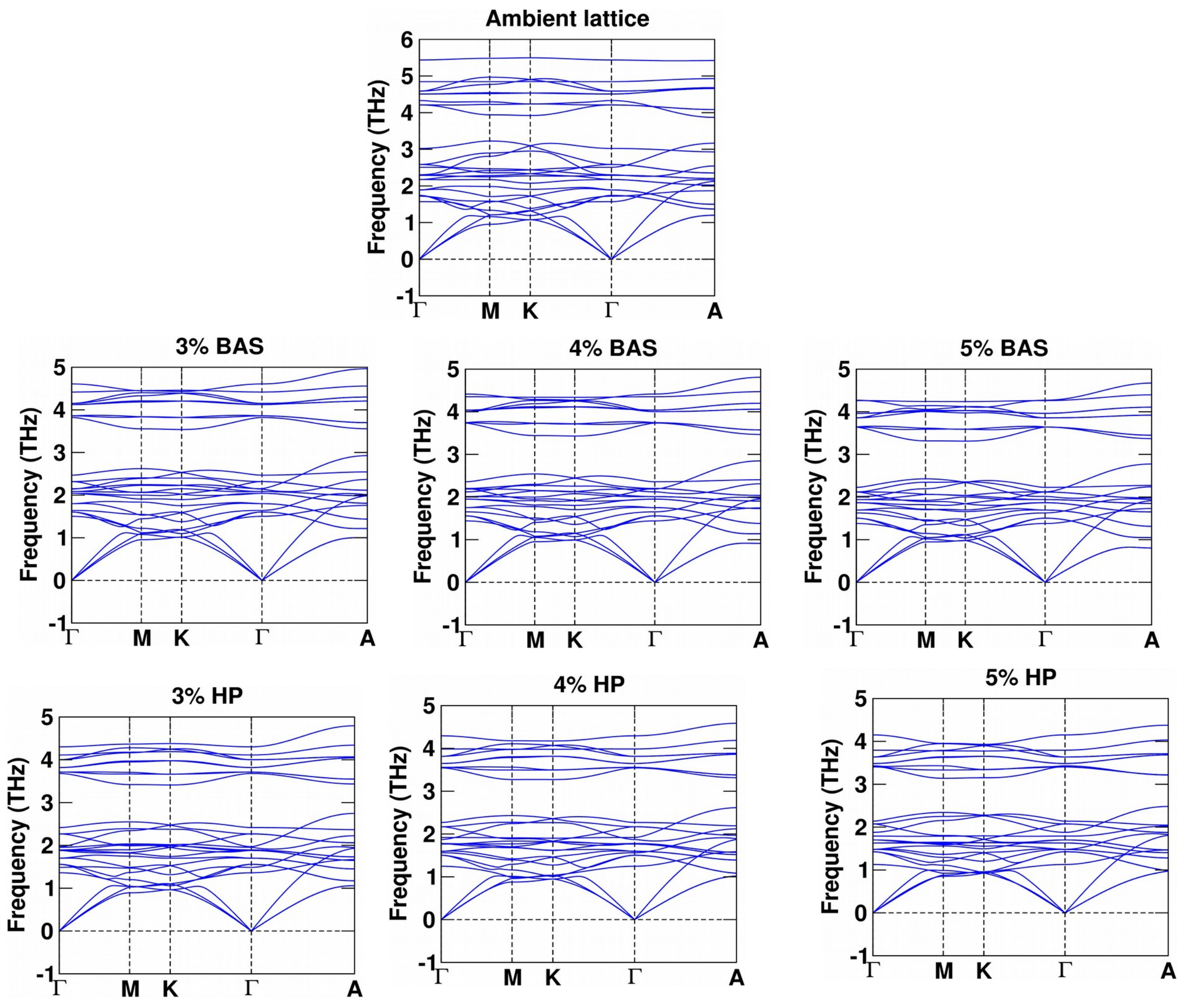

Fig. S2. Same as Fig.S1 but for NaSrBi

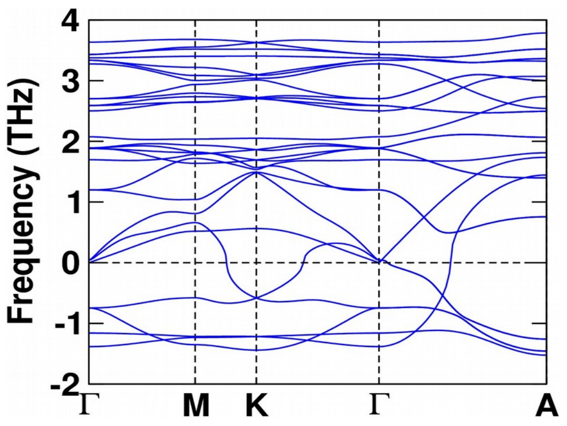

Fig. S3. For phonon dispersion of RbCaBi at ambient lattice constatnt

III. Surface dispersions of NaCaBi and NaSrBi using PBE exchange correlation functional

In the main manuscript, we have discussed the non-trivial band topology of NaCaBi and NaSrBi using PBE exchange correlation functional. The bulk electronic structure is shown in Fig. 1 (c-f) of main manuscript. Here, we have provided corresponding surface spectra. Fig. S4 shows the surface electronic structure for both the compounds.

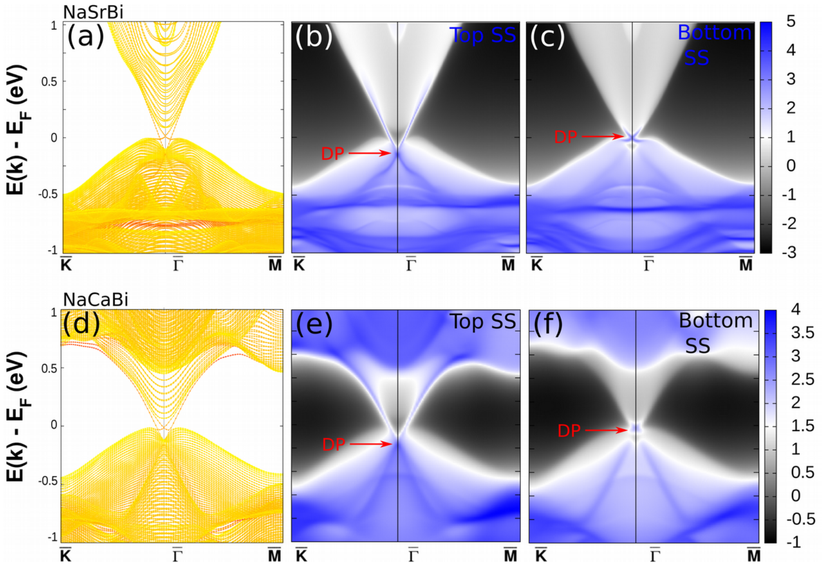

Fig. S4. (a) Surface dispersion and (b,c) surface density of states for top and bottom surface layers for NaSrBi. (d-f) shows the similar plots for NaCaBi.

IV. Analysis of topological properties for NaCaBi and NaSrBi under Hydrostatic Pressure

In this section, we discuss the effect of hydrostatic pressure on the non-trivial band topology. Using HSE06 exchange correlation functional, we found trivial band ordering for both NaSrBi and NaCaBi at their respective ambient conditions. Therefor, we have applied hydrostatic pressure (HP) in terms of lattice expansion and checked the evolution of bands topology around the Fermi level at Γ point. A trivial to non-trivial phase transition occurs at ~ - 2 GPa (around 1% expansion in lattice parameter) and both the materials hold their non-trivial band ordering at higher pressures as well. Here, we studied the topological properties of both the systems at 3% HP. Fig. S5 shows the bulk electronic structure of both the systems at 3% HP using PBE and HSE06 exchange correlation functional. The calculated electronic structures using PBE and HSE06 show similar band ordering for both the systems. Hence it is reasonable to expect similar surface dispersion at PBE and HSE06 level of calculations. We, therefore, took the PBE functional to construct the maximally localized Wannier functions (MLWFs) from Na-s, Sr/Ca-s orbital and Bi-pz orbitals. Fig. S6 shows the surface band dispersion and surface density of states for NaSrBi

(top panel, a-c) and NaCaBi (bottom panel, d-f). Since the slab calculation involves two surfaces, the corresponding surface bands and spectral intensity maps for both the surfaces (top and bottom) are given.

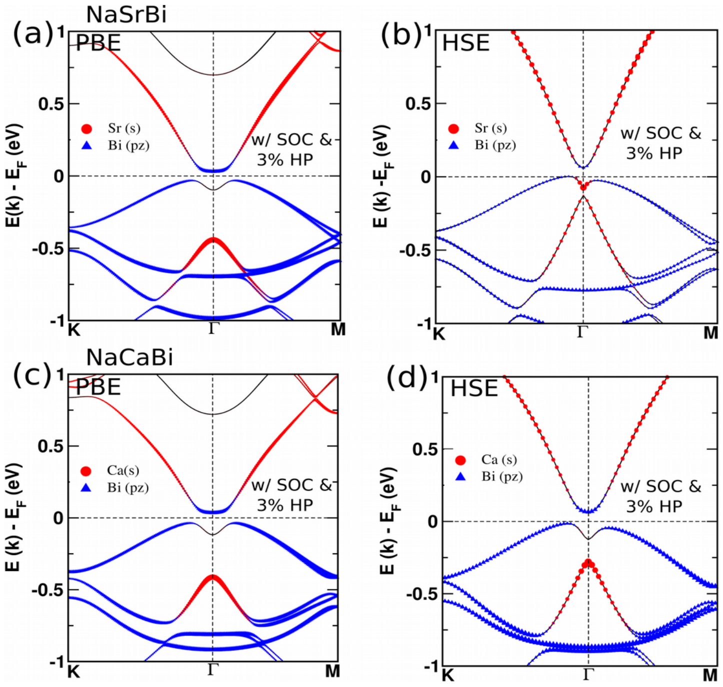

Fig. S5. Band structures using PBE and HSE06 functionals at 3% HP in the presence of SOC (a & b) for NaSrBi and (c & d) for NaCaBi.

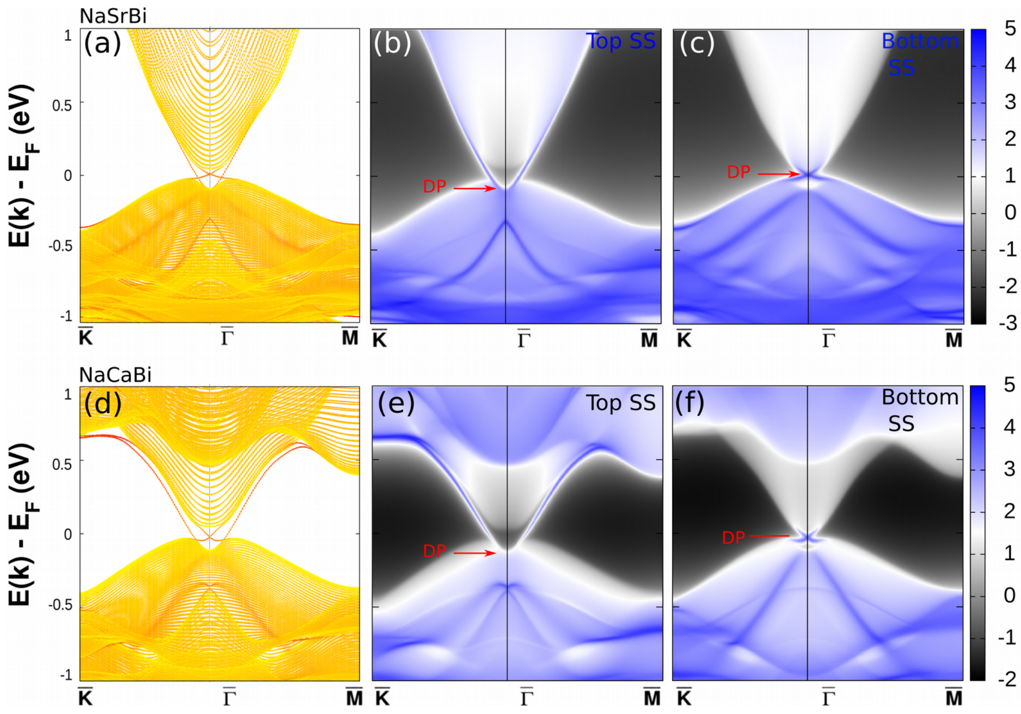

Fig. S6. (a) Surface dispersion and (b,c) surface density of states for top and bottom layers for NaSrBi at 3% HP. (d-f) shows the similar plot for NaCaBi.

V. Effects of compressive pressure in NaSrBi towards d-p band inversion

Here, we have discussed the effect of compression in NaSrBi to observe the band evolution. Our calculation show that a large amount of compression (~ 25 GPa) gives non-trivial band ordering. Fig. S7 shows band structure of NaSrBi at 14.5, 19.7 and 25.6 GPa compressive pressure. At around 25.6 Gpa (12% of lattice compression) a p-d band inversion occurs between Sr - dz^2 and Bi - pz orbitals. The lattice compression shift the Sr - s orbital towards higher energy where as Sr - d orbital pushed down to realize band inversion between Sr - dz^2 and Bi - pz as shown in the Fig. S7(c). The local environment of Sr and Na atom in NaSrBi is octahedral and tetrahedral which is distorted by Bi atoms. Application of compressive pressure increase the distortion of Sr octahedral which interns stabilize the Sr - dz^2 more. In the same time due to increase of coulomb repulsion between Sr^{2+} state and Bi^{3-} in the tetrahedral site, Sr - s orbital destabilize and shift towards higher energy. Therefor, comparative to p-d band inversion, it is much easier to get s-p band inversion by lattice expansion (around 1% of lattice expansion) which is explicitly studied in the main manuscript.

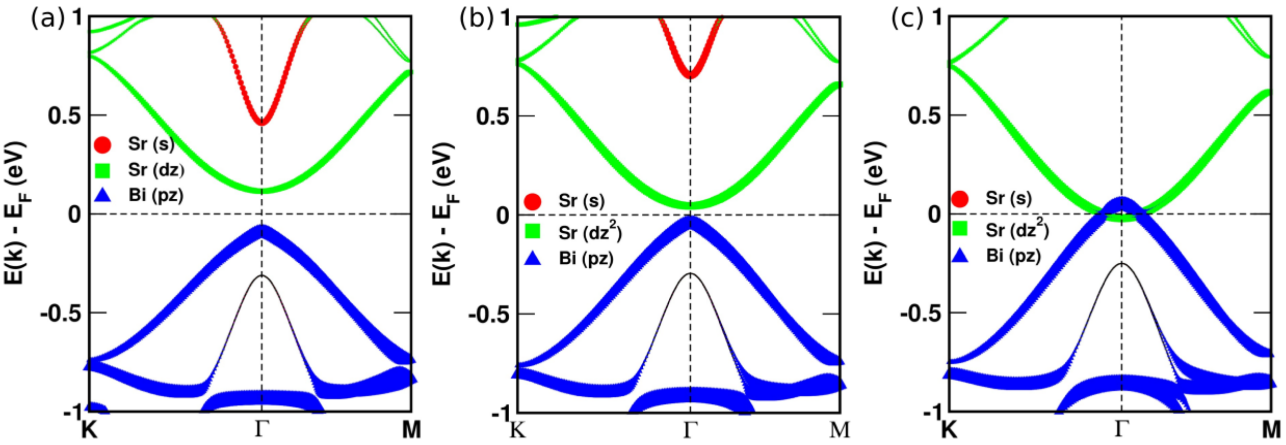

Fig. S7. PBE bulk electronic structure of NaSrBi with SOC at (a) 14.5 GPa (b) 19.7 GPa and (c) 25.6 GPa compressive pressure.

VI. Analysis of topological properties for NaCaBi and NaSrBi under bi-axial strain

We have explicitly discussed about the effect of bi-axial strain (BAS) towards the non-trivial band topology in the main manuscript within HSE06 level of calculation. We considered 3% BAS for detailed analysis on electronic structure and surface sprectra for both NaSrBi and NaCaBi compounds. The bulk electronic structure using GGA correlation is shown in the Fig. S8. It shows that the band ordering is similar at GGA and HSE06 level of calculations. Therefore, we took the GGA functional to construct the MLWFs and further simulated the surface dispersions for TI phases of NaSrBi and NaCaBi at +3% BAS along [110].

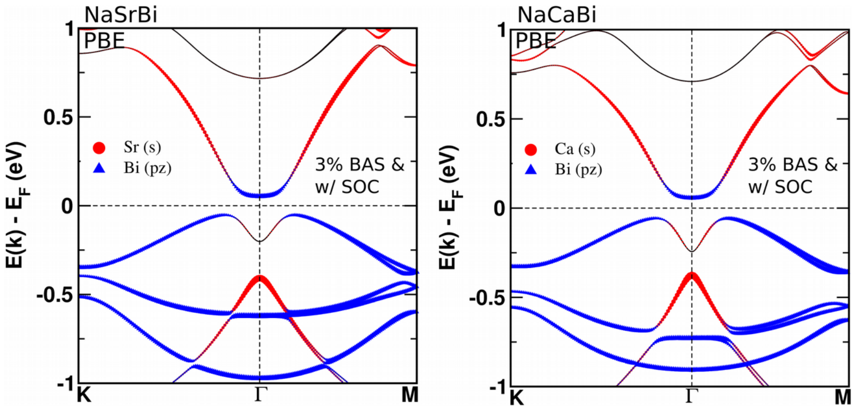

Fig S8. GGA bulk electronic structure of NaSrBi and NaCaBi under 3% bi-axial strain.

VII. Doping effect towards non-trivial band topology

Hydrostatic pressure can be realized by compressing/expanding the lattice constants as discussed in the previous section. Doping with bigger atoms could be another way to achieve this. As such, we have doped Na by Rb/Cs in the parent compounds NaSrBi and NaCaBi. We have studied the HSE06 band topology replacing one Na atom by one X=Rb\Cs atom in the unit cell, giving $Na_{0.66}X_{0.33}CaBi$ and $Na_{0.66}X_{0.33}SrBi$ compounds. Fig. S9 shows the bulk band structures of $Na_{0.66}Rb_{0.33}CaBi$ and $Na_{0.66}Cs_{0.33}CaBi$ alloys. Due to the doping by large sized atoms, the lattice expands and gives a band inversion naturally instead of an external pressure. It is clear from the Fig. S9 that the non-trivial band gap increases for higher doping concentrations.

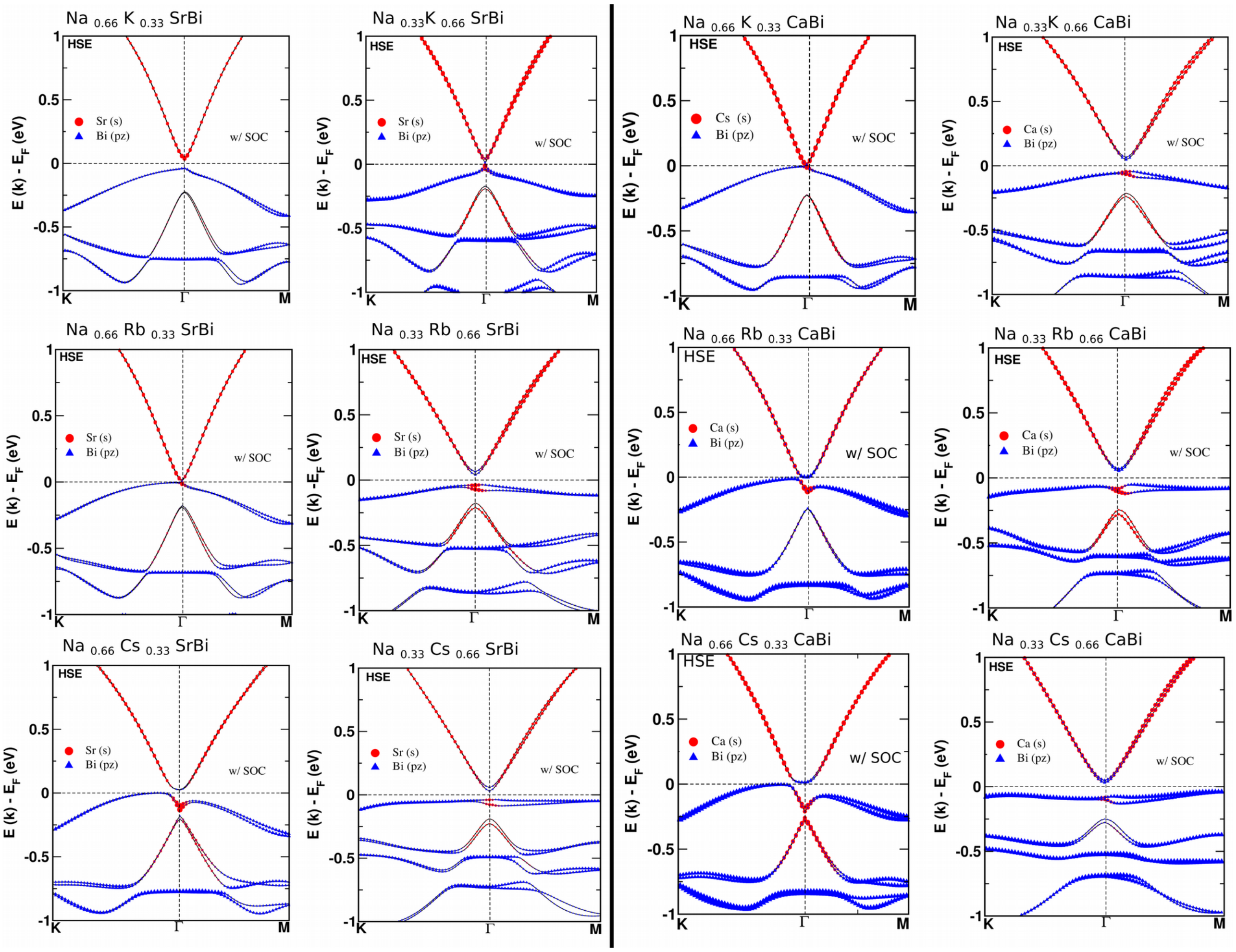

Fig. S9. Band structures using HSE06 functional in the presence of SOC. Left and right panel for NaSrBi and NaCaBi respectively with 33% and 66% doping concentrations.

VIII. Topological phase in RbSrBi, CsCaBi and RbBaBi

Here we have studied the topological phases of CsSrBi, CsCaBi and RbSrBi. Similar to RbCaBi (explicitly discussed in main manuscript Fig. 5.), these there compounds show topological nodal line semi-metal states in the absence of SOC. Inclusion of SOC opens a small gap along the nodal lines and the compounds converted to small gap topological insulator. In case of CsSrBi and CsCaBi, although it open up a small gap, the dispersion is still like NLS and expected to realize the torus shape bulk Fermi surface and topological drum head like topological surface states .

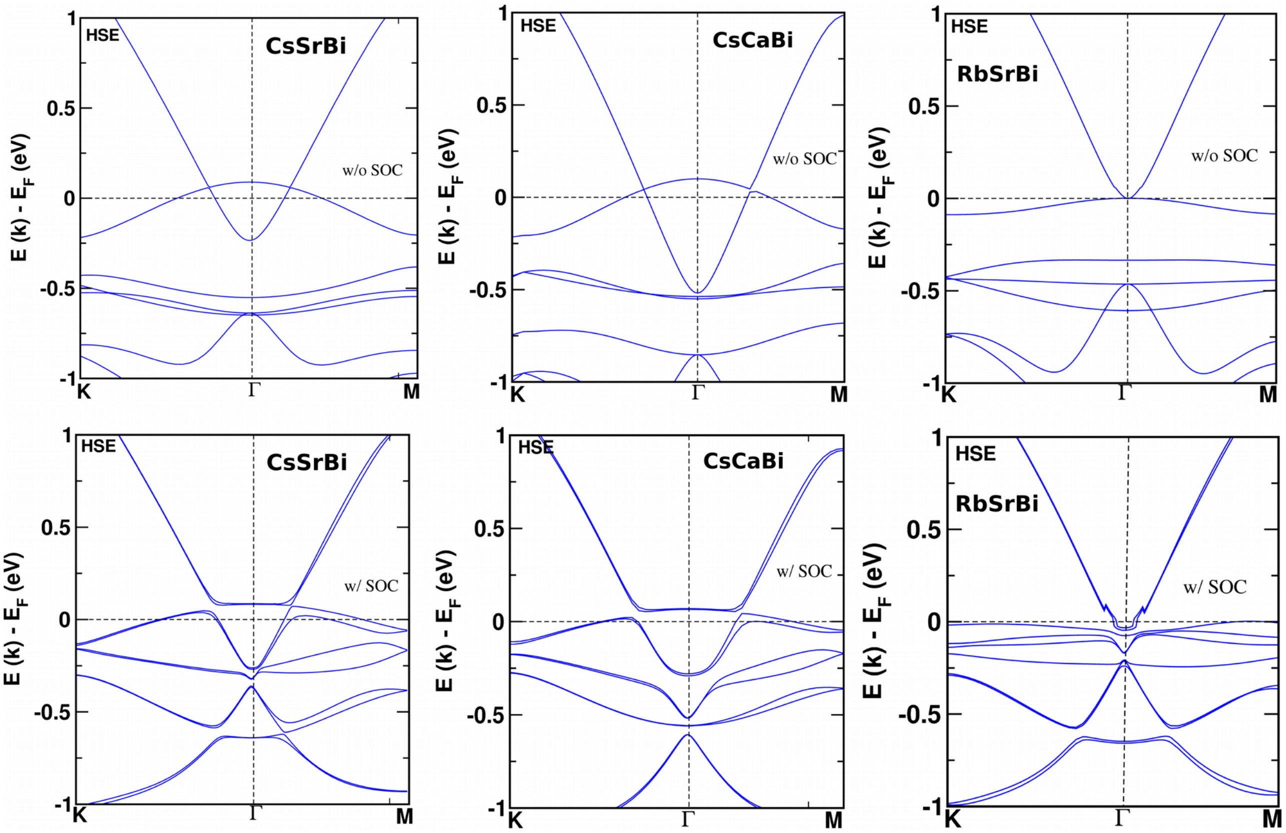

Fig. S10. HSE06 bulk electronic structures of CsSrBi, CsCaBi and RbSrBi. Top panel without SOC and bottom panel with SOC.

IX. Band structures in full BZ (using GGA) and Γ-A direction (using HSE06)

Topological insulating property is a global property of bulk band structure. To confirm the insulating nature with no band crossing across the Fermi level, we have plotted the band structure of our predicted TIs in full BZ using GGA approximation, as shown in Fig. S11(a) and S12(a) for NaCaBi and NaSrBi respectively. As Fermi crossing is often observed along $\Gamma - A$ path in the hexagonal compounds, we further cross checked by doing a more accurate HSE06 calculation along $\Gamma - A$ direction to assure the absence of band gap clossing in our predicted compounds. HSE06 calculations are also done for the strain cases, as shown in Fig. S11(c,d) and S12(c,d) for the two compounds. All these calculations indeed show finite non-trivial band gap at/around Γ point and no crossing of bands cross the Fermi level in the whole Brillouin zone.

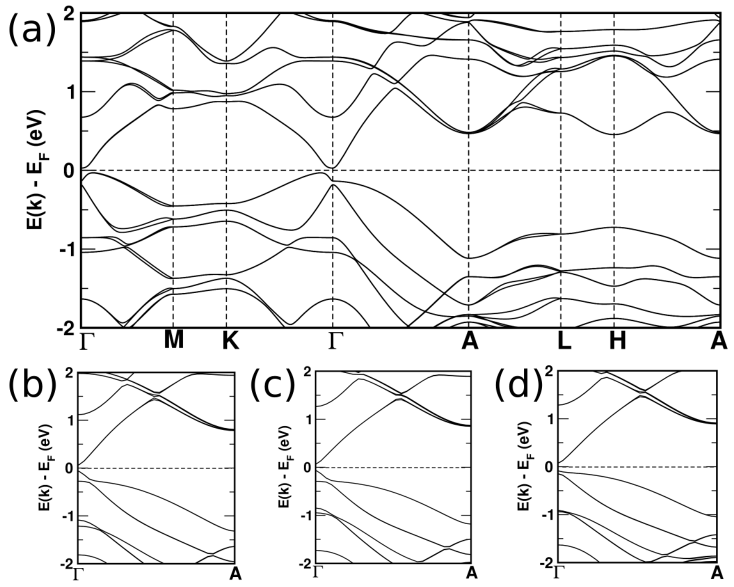

Fig. S11. (a) Balk band structure of NaCaBi using GGA functional in full BZ. (b-d) are the band structures along Γ-A direction using HSE06 functional at ambient condition, 3 % BAS and 3 % HP respectively.

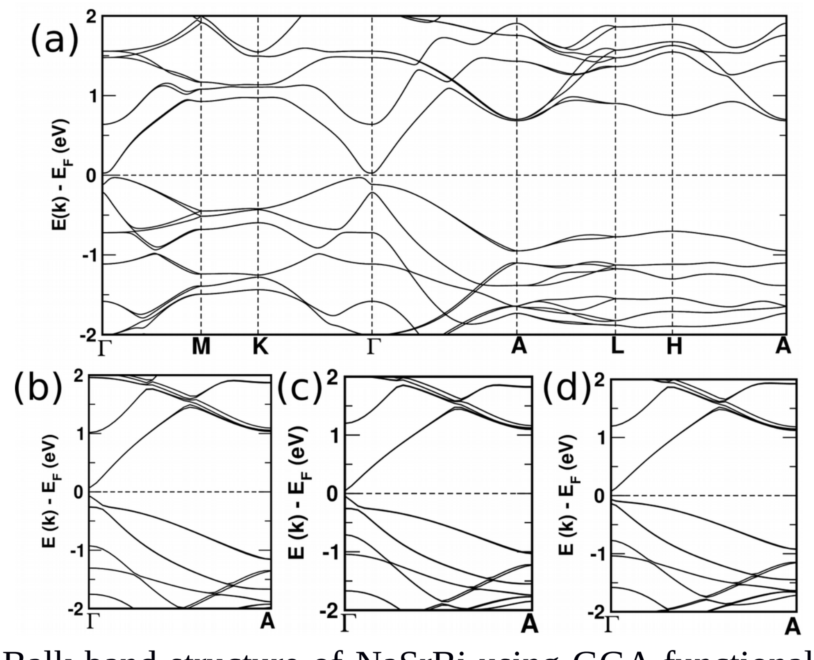

Fig. S12. Balk band structure of NaSrBi using GGA functional in full BZ. (b-d) are the band structures along Γ-A direction using HSE06 functional at ambient condition, 3 % BAS and 3 % HP respectively.

References

1. E. Lora da Silva et al., Phys. Rev. B 91, 144107 (2015).

2. Arthur Marronnier et al., J. Phys. Chem. Lett. 8, 2659 (2017).

3. Federico Brivio et al., Phys. Rev. B 92, 144308 (2015).

4. Jianfeng Yang et al., Nat. Commun. 8, 14120 (2017).

5. Christopher E. Patrick et al., Phys. Rev. B 92, 201205(R) (2015).

6. Lucy D. Whalley et al., Phys. Rev. B 94, 220301(R) (2016).
